# Editorial: Advances in artificial intelligence (AI) in brain computer interface (BCI) and Industry 4.0 for human machine interaction (HMI)

**DOI:** 10.3389/fnhum.2023.1320536

**Published:** 2023-12-05

**Authors:** Umer Asgher, Yasar Ayaz, Redha Taiar

**Affiliations:** ^1^Quality Assurance & NUST International Office Directorate (QA & NIO Dte), National University of Sciences and Technology (NUST), Islamabad, Pakistan; ^2^National Center of Artificial Intelligence (NCAI), National University of Sciences and Technology (NUST), Islamabad, Pakistan; ^3^Laboratoire MATériaux et Ingénierie Mécanique (MATIM), Université de Reims Champagne-Ardenne, Reims, France

**Keywords:** advances in artificial intelligence, brain computer interface (BCI), Industry 4.0–advantages and challenges, human machine interaction, technologies

## Introduction

The convergence of Artificial Intelligence (AI) and Brain-Computer Interface (BCI) has witnessed substantial advancements, particularly in the domains of emotion recognition and cognitive screening. This comprehensive editorial delves into the latest developments presented in the Research Topic “*Advances in Artificial Intelligence (AI) in Brain-Computer Interface (BCI) and Industry 4.0 for Human-Machine Interaction (HMI)*” of Front. Hum. Neurosci., Sec. Brain-Computer Interfaces. Over the past decade, noteworthy industrial progress has transpired in computerized control and monitoring applications, further catalyzing the integration of advanced technologies such as BCIs empowered by artificial intelligence. Modern BCIs are situated at the confluence of data acquisition, signal processing, artificial intelligence, and cyber-physical systems (CPS). Innovations in algorithms, particularly in cognitive computing, are fueling the continuous infusion of artificial intelligence into realms like BCIs, Industry 4.0, and Surgery 4.0 (healthcare), with the aim of establishing a robust industrial artificial intelligence ecosystem. Industry 4.0, a swiftly evolving sector, seeks to revolutionize traditional industrial methods through the deployment of digital tools such as artificial intelligence and brain-computer interfaces. Sophisticated artificial intelligence algorithms, including machine and deep learning, play a pivotal role in enhancing the performance of a BCI system, facilitating more effective management of real-life challenges. BCI-based solutions are gaining traction in bolstering industrial performance, from precise assessment to optimizing neuroergonomic systems, accurately evaluating the mental and cognitive workload of industrial operators, facilitating human-robot interactions, robot-assisted surgeries, and ensuring safety in critical conditions.

BCIs offer a methodology for manipulating computers and external mechatronic devices based on brain signals. Recently, the modern industrial sector has exhibited a growing interest in BCI-operated machines. The research and development of innovative BCIs, coupled with advancements in AI, may eventually give rise to a robust artificial intelligence-centric industry. Studies suggest that BCIs may find applications outside of laboratories and in ecologically relevant settings. However, deploying BCI systems in real-world ecological applications poses various challenges, including the need for accurate recognition of human mental states and emotions. Addressing these challenges in emotion detection, mental workload, and mental state recognition may require sophisticated approaches based on novel machine or deep learning models. In modern industry and healthcare, efforts are underway to develop hardware, software, machines, and devices with human-like intelligence.

This issue serves as a platform to share ideas, approaches, opinions, and comments on the latest research in AI and BCI, emphasizing challenges pertinent to the future deployment of intelligent AI-based BCI applications for Industry 4.0. Additionally, it aims to provide a forum for researchers to investigate the role of these AI methods in enhancing the performance of existing BCI applications. Four papers are included in this Research Topic, comprising three original research papers and one review. The contributions of these papers underscore the significance of advanced technologies like AI-augmented BCIs in modernizing and improving the quality of life and related applications.

In the first original research paper, Liang et al. introduce two primary contributions. The first is a multi-source joint-domain adaptation network proposal that addresses the challenge of cross-domain generalization in electroencephalography (EEG) emotion recognition. The second involves extensive cross-subject and cross-session transfer experiments on a publicly available emotion EEG dataset, validating the effectiveness of the proposed method. The second original research paper, by Ran et al., presents three key contributions. First, the authors propose a simplified style transfer mapping method based on the instance selection (SSTM-IS) algorithm, which enhances the accuracy and speed of cross-subject emotion recognition by selecting informative instances and simplifying the update strategy of hyperparameters in style transfer mapping. Second, the proposed algorithm is validated on both public and self-collected datasets, demonstrating higher accuracy in a shorter computing time for real-time emotion recognition applications. Third, the authors design and implement a real-time emotion recognition system that integrates EEG signal acquisition, data processing, emotion recognition, and result visualization. In the third original research paper, Li et al. introduce a novel model, STGATE, which combines a Transformer Learning Block (TLB) and a Spatial-Temporal Graph Attention (STGAT) mechanism. The TLB leverages 2D convolutional layers and a transformer encoder to extract time-frequency information, while the STGAT incorporates spatial and temporal attention mechanisms to capture connections between brain regions and temporal information. The authors' approach treats EEG signals as graph data and integrates them into graph neural networks to capture correlations between EEG channels. In their review, Sirilertmekasakul et al. focus on the application of three AI implementations: machine learning (ML) and deep learning (DL), computer vision, and automatic speech recognition (ASR). The authors discuss the advantages and limitations of digitized cognitive screening tests.

## Conclusion

The Research Topic “*Advances in Artificial Intelligence (AI) in Brain-Computer Interface (BCI) and Industry 4.0 for Human-Machine Interaction (HMI)*” collectively signifies the dynamic evolution of AI-BCI applications, ranging from real-time emotion recognition to the digitization of cognitive screening tests. These advancements not only contribute to understanding human emotions through EEG signals but also pave the way for accessible and efficient cognitive assessments. The interdisciplinary nature of these studies underscores the potential of AI-BCI in shaping the future of healthcare and human-machine interaction. This Research Topic serves as an invaluable resource for readers seeking in-depth knowledge and understanding of the intricacies of these modern technologies and their significance in contemporary life.

## Author contributions

UA: Conceptualization, Investigation, Writing – original draft. YA: Investigation, Validation, Visualization, Conceptualization, Writing – original draft. RT: Writing – original draft, Writing – review & editing.

